# Short-term cigarette smoke exposure aggravates oxidative stress and airway inflammation induced by lipopolysaccharides

**DOI:** 10.3389/fphys.2026.1788828

**Published:** 2026-03-19

**Authors:** Ziyao Liang, Zhihang Liu, Wenchao Pan, Long Fan, Jingyu Quan, Lin Lin, Lei Wu, Xuhua Yu

**Affiliations:** State Key Laboratory of Traditional Chinese Medicine Syndrome, The Second Affiliated Hospital of Guangzhou University of Chinese Medicine/Guangzhou University of Chinese Medicine, Guangzhou, Guangdong, China

**Keywords:** cigarette smoke, inflammation, LPS, oxidative stress, airway remodeling

## Abstract

**Aim:**

This study discloses the early synergistic effects of short-term cigarette smoke (CS) exposure combined with lipopolysaccharide (LPS) on pulmonary inflammation and tissue stress.

**Method:**

Six- to eight-week-old BALB/c mice were divided into CS-exposed groups (9 cigarettes per day for 4 days) and sham-exposed control groups. On the fourth day, intratracheal instillation of LPS or saline was administered to both groups. The study examined several indicators, including changes in body weight, bronchoalveolar lavage fluid (BALF) cell counts, mRNA expression of inflammatory factors and oxidative stress markers, lung histopathology, and airway remodeling markers.

**Result:**

The results showed that short-term CS exposure alone did not induce significant oxidative stress or inflammation. However, short-term CS exposure exacerbated LPS-induced pulmonary inflammation, as evidenced by increased expression of pro-inflammatory cytokines, including IL-6, IL-1β, and TNF-α. It also intensified oxidative stress, as indicated by upregulation of NADPH oxidase 2 (NOX2) and heme oxygenase-1 (HO-1). Additionally, activation of early airway remodeling–associated signaling was observed, with elevated expression of collagen I/III, alpha-smooth muscle actin (α-SMA), and transforming growth factor-β1 (TGF-β1). These effects occurred through activation of NF-κB-mediated inflammatory pathways, increased macrophage-derived reactive oxygen species (ROS) production, and reduced antioxidant defenses. Notably, short-term CS exposure did not significantly affect the number of immune cells in BALF after LPS stimulation.

**Conclusion:**

These results indicate that short-term CS exposure can “sensitize” the lungs—that is, increase their sensitivity—to acute lung injury upon subsequent bacterial stimulation. These findings suggest that even brief CS exposure may increase sensitivity to infection-associated acute lung injury in passive or intermittent smokers.

## Introduction

Pulmonary exposure to harmful environmental agents and microbial components significantly contributes to the development of both acute and chronic respiratory diseases. Among these agents, cigarette smoke (CS) is a major factor in respiratory morbidity and mortality worldwide (Gan et al., 2022; World Health Organization, 2023). CS is a complex mixture containing over 7,000 chemicals (CDC, 2024), many of which possess oxidant, carcinogenic, and immunomodulatory properties. It is well known for its role in the development of chronic obstructive pulmonary disease (COPD), emphysema, and bronchitis, primarily through mechanisms such as sustained inflammation, oxidative stress, an imbalance between proteases and antiproteases, and structural remodeling of lung tissue (Hill et al., 2000; Zhou et al., 2020; Liu et al., 2023; Van Eeckhoutte et al., 2023; Chen et al., 2024).

A considerable body of research has focused on the long-term effects of CS exposure, which has been modeled to understand its chronic impact on lung structure and function (Churg et al., 2011; Barnes, 2020; Chen et al., 2024; Wohnhaas et al., 2024). These studies have demonstrated that prolonged exposure leads to persistent activation of inflammatory signaling pathways, such as nuclear factor kappa-B (NF-κB), recruitment of immune cells, including neutrophils and macrophages, induction of matrix metalloproteinases, and a decline in antioxidant defenses. However, short-term exposure to cigarette smoke, although less frequently examined, is not without consequences. Even brief exposure can disrupt epithelial barrier integrity, create oxidative imbalances, and alter immune signaling, potentially increasing lung sensitivity to subsequent damage. This is particularly important for individuals who smoke intermittently, are exposed to secondhand smoke, or experience early exposure before the onset of chronic disease (Doz et al., 2008; Sakhatskyy et al., 2017; Estur et al., 2024; Seo et al., 2024). Concurrently, lipopolysaccharide (LPS), a well-known pathogen-associated molecular pattern derived from the outer membrane of Gram-negative bacteria, has been extensively used to study acute lung inflammation (Chen et al., 2010; Xiang and Fan, 2010; Domscheit et al., 2020). When delivered to the lung, LPS activates Toll-like receptor 4 (TLR4) on epithelial and innate immune cells, triggering the rapid release of pro-inflammatory cytokines such as interleukin-1β (IL-1β), tumor necrosis factor-α (TNF-α), and interleukin-6 (IL-6), as well as inducing neutrophil-attracting chemokines such as CXCL1 and CXCL2, which promote neutrophil recruitment. The LPS model recapitulates several hallmark features of acute lung inflammation (ALI) and is widely used to investigate pathways relevant to acute respiratory distress syndrome (ARDS) and bacterial pneumonia.

It is important to note that in real-world scenarios, cigarette smoke and lipopolysaccharide (LPS) are not isolated factors. Individuals who smoke or are exposed to cigarette smoke face an increased risk of pulmonary infections. Smoking has been linked to altered airway microbial communities and colonization by respiratory pathogens, which may contribute to infection-driven exacerbations (Charlson et al., 2010; McGeoch et al., 2023). In these contexts, cigarette smoke exposure can impair multiple host defense mechanisms that are directly relevant to bacterial products such as LPS. In smoking-related airway disease, impaired mucociliary clearance and epithelial injury, combined with dysregulated innate immune responses—including macrophage dysfunction and neutrophil-dominant inflammation—are considered key contributors to susceptibility to bacterial colonization and infection-driven exacerbations (Sethi and Murphy, 2008; Christenson et al., 2022). Accordingly, experimental “double-hit” studies—including controlled human LPS inhalation—have begun to explore how cigarette smoke exposure influences LPS-driven lung inflammation and injury. These studies support the idea that smoke pre-exposure can prime the lung microenvironment and modify subsequent inflammatory responses after LPS challenge (Borgas et al., 2016; Moazed et al., 2016; Sakhatskyy et al., 2017).

However, a significant gap still exists in understanding how short-term cigarette smoke exposure affects the immediate pulmonary response to LPS. This issue is especially important because even brief smoke exposure can prime the lung’s immune and oxidative environments, worsening responses to later endotoxin challenges (Gotts et al., 2017; Seo et al., 2024). Gaining insight into these early interactions is vital, especially regarding acute infections in individuals recently exposed to smoke or in populations with intermittent exposure, such as adolescents, passive smokers, or those in high-risk occupational settings. In this study, we aim to address this knowledge gap by exploring the early synergistic effects of short-term cigarette smoke exposure combined with LPS instillation on pulmonary inflammation and tissue stress. By focusing on the initial interactions between these two common stressors, we seek to clarify the molecular and cellular mechanisms underlying acute inflammatory exacerbations. Such insights may be particularly relevant to populations at elevated risk.

## Methods

### Animals

Specific pathogen-free male BALB/c mice, aged 6–8 weeks, were obtained from the Guangdong Medical Laboratory Animal Center and randomly assigned to four groups: sham + vehicle, CS + vehicle, sham + LPS, and CS + LPS (sham: sham-exposed control; CS: cigarette smoke exposure; vehicle: saline; LPS: lipopolysaccharide). All animals were housed under standard conditions (23 °C ± 2 °C, 12-h light/dark cycle) with *ad libitum* access to food and water. All animal procedures were approved by the Institutional Animal Care and Use Committee of the Guangdong Provincial Academy of Chinese Medical Sciences (protocol number 2023112).

### Cigarette smoke exposure

Mice were exposed to cigarette smoke (CS) in a 14-L plastic chamber placed inside a fume hood. CS was generated from nine cigarettes per day for four consecutive days (Days 0–3), following published short-term exposure paradigms (Ferreira et al., 2014; Seo et al., 2024). Exposure was administered three times daily (three cigarettes per session for 1 hour) at approximately 2-h intervals. Sham-exposed control mice were housed in an identical chamber without CS exposure. Filter-tipped DaQianMen cigarettes (Shanghai Tobacco Group Co., Ltd., China) were used, each containing 13 mg of CO, 0.8 mg of nicotine, and 11 mg of tar. Mice were weighed daily at a consistent time.

### Lipopolysaccharide intratracheal instillation

The mice were anesthetized via intraperitoneal injection of pentobarbital sodium (Virbac, NSW, Australia) at a dose of 100 mg/kg, followed by intratracheal instillation of either 10 μg of lipopolysaccharide (LPS) from *Escherichia coli* O55:B5 (Sigma-Aldrich, United States) dissolved in 50 µL of saline, or an equivalent volume of saline alone on day 4. The LPS dose was selected based on well-established murine models of acute lung inflammation, demonstrating that this dose reliably induces a reproducible neutrophilic inflammatory response and acute lung injury (Asti et al., 2000; Du et al., 2018; Schwab et al., 2024).

### Bronchoalveolar lavage and lung collection

The mice were euthanized by intraperitoneal injection of pentobarbital sodium (Virbac, NSW, Australia) at an overdose dose of 250 mg/kg, followed by cervical dislocation on day 5. Subsequently, the lungs were rinsed *in situ* with 0.4 mL of a solution containing 0.2% fetal bovine serum (FBS; Gibco, United States) in phosphate-buffered saline (PBS; Gibco, United States). Three additional rinses with 0.3 mL of PBS were performed, yielding approximately 1 mL of bronchoalveolar lavage fluid (BALF) per mouse. A 50 µL aliquot of BALF was collected for a cellular oxidative stress assay.

The viable cell count of the collected BALF was determined, and cytospin slides were prepared. Differential cell counts were performed using standard morphological criteria. The remaining BALF was then centrifuged to separate the supernatant, which was stored at −80 °C. The whole lungs were perfused via the right ventricle with 5 mL of saline to remove intravascular cells. After rinsing with saline, the right middle lobe was immersed in 4% paraformaldehyde solution for paraffin embedding, while the remaining lung tissue was rapidly frozen in liquid nitrogen and stored at −80 °C.

### Detection of superoxide in bronchoalveolar lavage cells

For basal measurements, bronchoalveolar lavage (BAL) cells (50 µL of BAL fluid) were exposed to the chemiluminescent probe L-012 (100 μM; Wako Chemicals, Japan). In the presence of the protein kinase C (PKC) and NADPH oxidase activator phorbol 12,13-dibutyrate (PDBu, 1 μM; Sigma-Aldrich, United States), the cells were dispensed into a 96-well plate for luminescence detection using the Multiscan Spectrum (Tecan Infinite M1000pro, Austria). Photon emission was recorded from each well for 1 s, and the process was repeated 60 times. Individual data points for each group were obtained by averaging the results from two replicates. These values were then corrected by subtracting the average background values and normalized to total cell numbers. The results were expressed as relative light units (RLU).

### Flow cytometry analysis of bronchoalveolar lavage cells

The lavage fluid was collected as described above and centrifuged for 5 min at 400 × g and 4 °C. The cell pellet was resuspended in 200 µL of ACK lysing buffer (Boster, China) to lyse erythrocytes. Subsequently, 900 µL of cold DPBS was added to dilute the lysing buffer, followed by immediate centrifugation and removal of the supernatant. After assessing the single-cell suspension using trypan blue, the cells were resuspended in 0.1 mL of DPBS containing 0.2% FBS at a density of 1 × 10^6^ cells per 0.1 mL. The cells were then stained with fluorophore-conjugated antibodies targeting specific surface markers to identify immune cells (the antibodies and dilution ratios used for immune cell phenotyping are listed in Supplementary Table S1). For superoxide detection, the Cellular ROS/Superoxide Detection Assay Kit (Abcam, United States, cat# ab139476) was used according to the manufacturer’s instructions. After staining, cells were stimulated with phorbol 12,13-dibutyrate (PDBu, 1 μM; Sigma-Aldrich, United States) before analysis.

### Quantitative real-time PCR (qPCR)

Approximately 40 mg of whole lung tissue from each mouse was used to isolate total RNA. RNA extraction was performed using NucleoZOL reagent (Macherey-Nagel, Germany), followed by reverse transcription to cDNA with PrimeScript™ RT Master Mix (TaKaRa, Japan). Quantitative real-time PCR was conducted on an ABI QuantStudio 7 Flex system using TB Green® Premix Ex Taq™ II (Tli RNaseH Plus) (TaKaRa, Japan). Relative gene expression was calculated using the 2^−ΔΔCt method. Primer sequences used in this study are provided in Supplementary Table S2.

### Glutathione analysis

Glutathione (GSH) levels were measured using the Glutathione Fluorometric Assay Kit (BioVision, United States of America, cat# K264) according to the manufacturer’s protocol. Briefly, lung tissue (40 mg) was homogenized in 6 N perchloric acid and centrifuged at 13,000 × g for 2 min at 4 °C. The supernatant was neutralized with potassium hydroxide and centrifuged again to remove precipitates. GSH content was determined by incubation with an o-phthalaldehyde probe, and fluorescence was measured using a Multiscan Spectrum plate reader (Tecan Infinite M1000 Pro, Austria) at excitation and emission wavelengths of 340 nm and 420 nm, respectively.

### Hematoxylin and eosin (H&E) staining

Lung tissue was fixed in paraformaldehyde for 24 h, followed by dehydration through an ethanol gradient, clearing with xylene, and paraffin embedding. Tissue sections (4 μm) were deparaffinized and stained with hematoxylin and eosin (H&E) using a commercial kit (Servicebio, China, cat# G1076). For each animal, five non-overlapping fields were examined at 200× magnification under a light microscope, avoiding large blood vessels and major airways. Representative images from five randomly selected fields per animal were selected to reflect the group’s overall appearance. Histopathological assessment was performed by investigators blinded to the experimental group, focusing on features such as inflammatory cell infiltration and alveolar septal thickening.

### Terminal deoxynucleotidyl transferase dUTP nick end labeling (TUNEL) assay

Lung tissue sections were deparaffinized in xylene and rehydrated through a graded ethanol series. Antigen retrieval was performed by incubating the sections with Proteinase K solution (Servicebio, China) at 37 °C for 25 min. TUNEL staining was conducted using the CF488 TUNEL Cell Apoptosis Detection Kit (Servicebio, China, cat# G1504) according to the manufacturer’s instructions. Sections were incubated in a humidified chamber at 37 °C for 2 h, then washed three times with PBS (pH 7.4). Nuclei were counterstained with DAPI solution (Servicebio, China) for 10 min at room temperature. The sections were then mounted with an anti-fade medium and imaged using a fluorescence microscope. The apoptosis rate was calculated using ImageJ software as the percentage of TUNEL-positive cells relative to the total number of cells. Quantification was performed by investigators blinded to the experimental group.

### Immunohistochemistry

Paraffin-embedded lung sections were dewaxed, rehydrated, and subjected to antigen retrieval using a microwave-heated retrieval solution. Endogenous peroxidase activity was blocked with 3% H_2_O_2_, and sections were incubated in 5% BSA. Primary antibodies against collagen I (Abcam, United States, cat# ab21286), collagen III (Abcam, United States, cat# ab7778), and α-SMA (Santa Cruz Biotechnology, United States, cat# sc-32251) were applied overnight at 4 °C, followed by incubation with HRP-conjugated secondary antibodies. Immunoreactivity was visualized using DAB substrate and counterstained with hematoxylin. Because positive collagen granules are distributed only around the airways and blood vessels, we randomly selected five images containing as many airways and blood vessels as possible under a 400× light microscope. Using Image Pro Plus 6.0 software, we then evaluated the ratio of the positive granule integrated optical density (IOD) (mean IOD × positive granule area) to the lung parenchymal area. All analyses were performed by investigators blinded to the experimental group.

### Western blot

Total protein was extracted from frozen lung tissue using lysis buffer supplemented with protease and phosphatase inhibitors. Protein concentrations were determined using a BCA assay kit. Equal amounts of protein were separated by SDS-PAGE and transferred onto PVDF membranes. After blocking, membranes were incubated overnight at 4 °C with primary antibodies against NOX2 (gp91^phox) (1:2000, BD Biosciences, United States, cat# 611415), HO-1 (1:2000, Abcam, United States, cat# ab13248), nuclear factor erythroid 2-related factor 2 (Nrf2) (1:1000, Cell Signaling Technology, United States, cat# 12721), caspase-3 (1:1000, Cell Signaling Technology, United States, cat# 9662), cleaved caspase-3 (1:1000, Cell Signaling Technology, United States, cat# 9661), IκBα (1:1000, Cell Signaling Technology, United States, cat# 4814), phosphorylated IκBα (p-IκBα) (1:1000, Cell Signaling Technology, United States, cat# 9246), GAPDH (1:1000, Cell Signaling Technology, United States, cat# 2118), TGFβ-1 (1:1000, Santa Cruz Biotechnology, United States, cat# sc-52893), and β-actin (1:1000, Santa Cruz Biotechnology, United States, cat# sc-81178). Following incubation with HRP-conjugated goat anti-rabbit or goat anti-mouse IgG secondary antibodies, immunoreactive bands were visualized using the Gel Doc™ EZ imaging system (Bio-Rad, United States). β-actin or GAPDH was used as the housekeeping protein.

### Statistics

Data are presented as the mean ± standard error of the mean (SEM). The sample size (n) represents the number of mice per group. Statistical analyses were performed using GraphPad Prism 9.0 (GraphPad Software, United States). For comparisons involving four independent groups, one-way analysis of variance (ANOVA) was used, followed by Tukey’s HSD test for *post hoc* pairwise comparisons. For comparisons between two independent groups, Welch’s t-test was applied. A p-value of less than 0.05 was considered statistically significant.

## Results

### Cigarette smoke exposure exacerbates weight loss induced by lipopolysaccharide challenge

**FIGURE 1:**
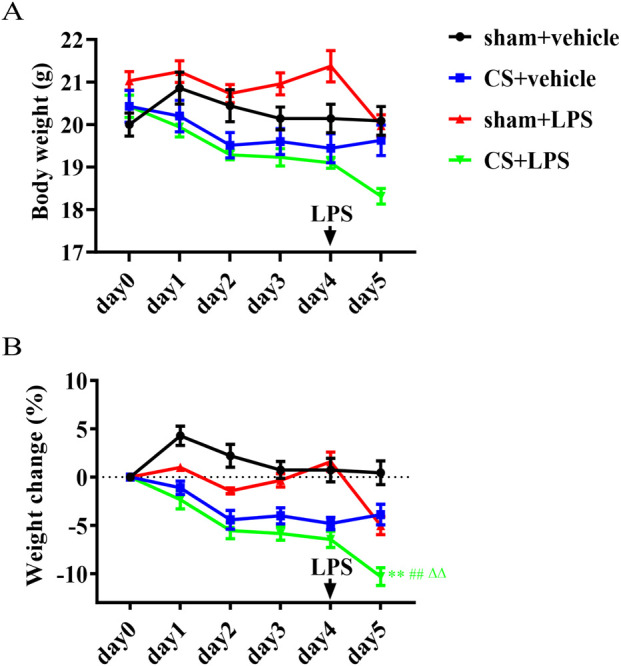
Cigarette smoke exposure exacerbates weight loss induced by lipopolysaccharide challenge. **(A)** The body weight of each mouse was measured daily. **(B)** The percentage change relative to day 0 was calculated. Mice were treated with LPS or vehicle via intratracheal instillation on day 4. Data are presented as mean ± SEM. n = 7. *P < 0.05, **P < 0.01, compared to sham + vehicle mice; △P < 0.05, △△P < 0.01, compared to CS + vehicle mice; and #P < 0.05, ##P < 0.01, compared to sham + LPS mice. Statistical significance was assessed using one-way ANOVA followed by Tukey’s HSD test, or Welch’s t-test as appropriate, as described in the Methods section.

Body weight was measured daily, and percentage changes from baseline values on day 0 were calculated (Figure 1). Baseline body weights on day 0 were comparable among groups (P > 0.05). Four days of CS exposure alone caused a 6.8% decrease in body weight compared to sham-exposed controls. Following LPS instillation, CS-exposed mice exhibited an additional 5.3% reduction in body weight compared to the sham + LPS group on day 5. This weight loss was significantly greater than that observed in the sham + vehicle, CS + vehicle, and sham + LPS groups (Figure 1B).

### Cigarette smoke exposure does not significantly affect bronchoalveolar lavage fluid cell counts following lipopolysaccharide challenge

**FIGURE 2:**
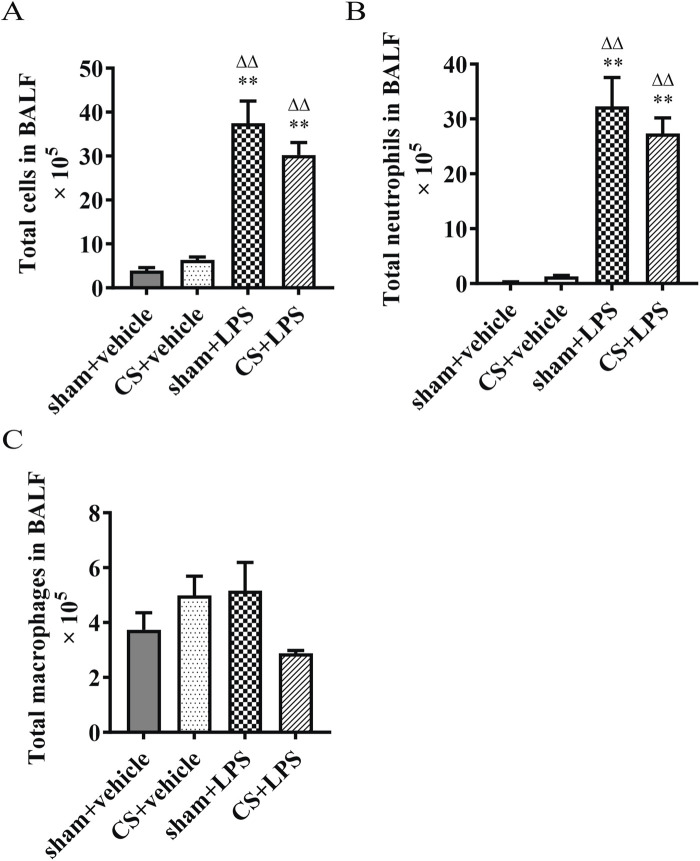
Cigarette smoke exposure does not significantly affect bronchoalveolar lavage fluid cell counts following lipopolysaccharide challenge. **(A)** Total cells. **(B)** Total neutrophils. **(C)** Total macrophages. Data are presented as mean ± SEM. n = 7. *P < 0.05, **P < 0.01, compared to sham + vehicle mice; △P < 0.05, △△P < 0.01, compared to CS + vehicle mice; #P < 0.05, ##P < 0.01, compared to sham + LPS mice.

The total and differential inflammatory cells in the BALF were quantified. As shown in Figure 2, LPS significantly increased the total number of cells and neutrophils in both sham-exposed and CS-exposed mice compared with vehicle-treated controls (P < 0.05). However, compared with the sham + LPS group, the CS + LPS group showed a modest decrease in total cells and neutrophils, although these differences did not reach statistical significance. Similarly, macrophage counts tended to be lower in CS + LPS mice than in sham + LPS mice, but the difference was not statistically significant (Figures 2A–C).

### Cigarette smoke exposure amplifies the expression of inflammatory genes induced by lipopolysaccharide challenge in lung tissue

To investigate the pulmonary inflammatory response, we measured the mRNA expression levels of key chemokines, pro-inflammatory cytokines, and proteases in whole lung tissue using quantitative real-time PCR (qPCR). As shown in Table 1, LPS significantly increased the expression of Cxcl1, Cxcl2, Cxcl5, MIP-1α, Il-6, Il-1β, Tnf-α, and Mmp9 in both sham-exposed and CS-exposed mice compared with vehicle-treated controls. Notably, CS exposure combined with LPS instillation further enhanced Il-6 (1.85-fold, p < 0.01), Il-1β (1.49-fold, p < 0.01), and Tnf-α (1.72-fold, p < 0.01) mRNA expression compared with LPS treatment alone.

**TABLE 1:** mRNA expression of cytokines, chemokines, and proteases in the whole lung.

*Gene*	Sham + vehicle	CS + vehicle	Sham + LPS	CS + LPS
*Chemokines*				
*Cxcl1*	1.17 ± 0.45	1.00 ± 0.17	14.66 ± 1.60^**△△^	15.24 ± 1.70^**△△^
*Cxcl2*	0.69 ± 0.17	1.02 ± 0.30	123.5 ± 17.94^**△△^	162.5 ± 29.16^**△△^
*Cxcl5*	0.87 ± 0.23	0.82 ± 0.13	26.36 ± 5.11^**△△^	23.08 ± 2.43^**△△^
*Mip-1α*	0.69 ± 0.15	0.91 ± 0.15	49.79 ± 5.78^**△△^	60.33 ± 5.58^**△△^
*Cytokines*				
*Il-6*	0.76 ± 0.21	1.16 ± 0.21	34.36 ± 3.42^**△△^	63.72 ± 5.70^**△△##^
*Il-1β*	1.00 ± 0.27	1.69 ± 0.16	20.98 ± 2.52^**△△^	31.20 ± 2.61^**△△#^
*Tnf-α*	0.73 ± 0.10	0.97 ± 0.19	18.19 ± 2.23^**△△^	31.34 ± 5.43^**△△#^
*M-csf2*	0.98 ± 0.12	1.52 ± 0.23	2.22 ± 0.59	1.86 ± 0.17
*Protease*				
*Mmp9*	0.99 ± 0.18	0.79 ± 0.08	3.79 ± 0.36^**△△^	4.04 ± 0.40^**△△^

Data are given as mean ± SEM. n = 6, 7, 7, 7, respectively, in group sham + vehicle, CS + vehicle, sham + LPS, CS + LPS. *P < 0.05, **P < 0.01, compared to sham + vehicle mice; △P < 0.05, △△P < 0.01, compared to CS + vehicle mice; #P < 0.05, ##P < 0.01, compared to sham + LPS mice.

### Cigarette smoke exposure exacerbates oxidative stress induced by lipopolysaccharide challenge in the lungs

**FIGURE 3:**
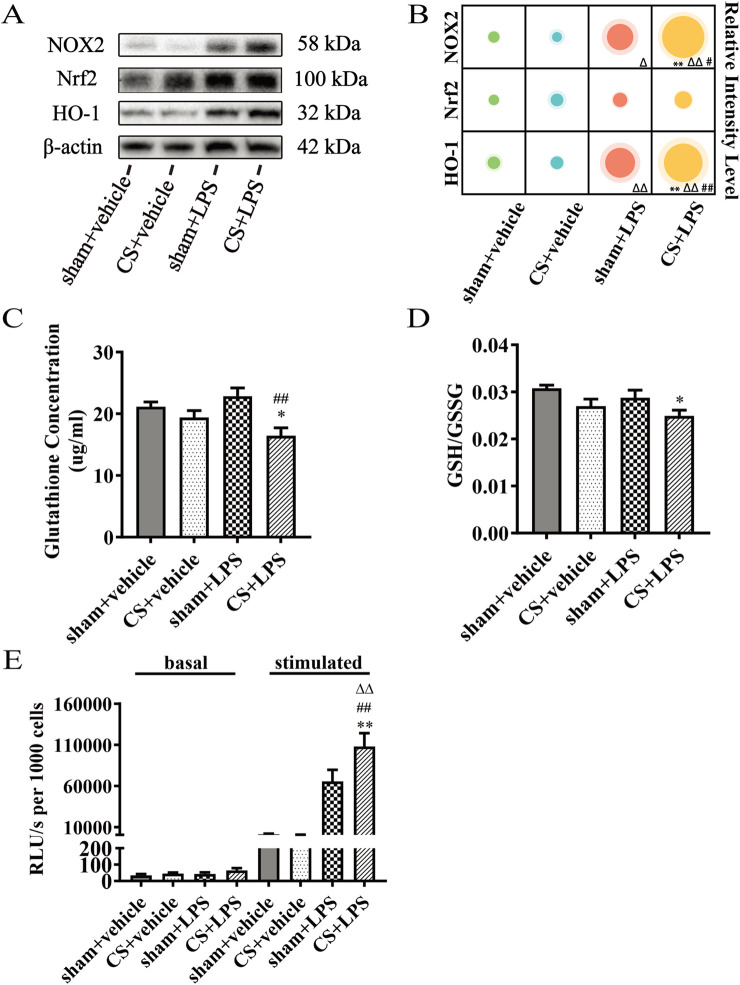
Cigarette smoke exposure amplifies the expression of inflammatory genes induced by lipopolysaccharide challenge in lung tissue. **(A)** Western blot analysis of protein levels of NOX2, Nrf2, and HO-1 in lung tissue. β-actin was used as the loading control. **(B)** Quantitative analysis of NOX2, Nrf2 and HO-1, n = 6. **(C)** Gutathione concentration, n = 7. **(D)** Ratio of GSH to GSSG, n = 7. **(E)** Superoxide production in BAL cells stimulated with PDBu was measured using the Multiscan Spectrum, n = 5–7. Data are presented as mean ± SEM. *P < 0.05, **P < 0.01, compared to sham + vehicle mice; △P < 0.05, △△P < 0.01, compared to CS + vehicle mice; #P < 0.05, ##P < 0.01, compared to sham + LPS mice.

To assess oxidative stress in the lungs, proteins associated with the Nrf2 signaling pathway were evaluated by Western blotting. As shown in Figures 3A,B, LPS instillation increased the protein expression of NOX2 and HO-1 in sham-exposed mice compared with sham + vehicle controls. Notably, CS exposure, when combined with LPS instillation, further enhanced NOX2 and HO-1 expression compared with LPS treatment alone. In contrast, Nrf2 protein levels did not differ significantly among the groups.

In addition, CS combined with LPS instillation significantly reduced pulmonary glutathione (GSH) levels and the GSH/glutathione disulfide (GSSG) ratio compared to sham + vehicle-treated controls. In contrast, LPS alone decreased GSH concentration in CS-exposed mice (Figures 3C,D). Superoxide production in bronchoalveolar lavage (BAL) cells was further assessed using the chemiluminescent probe L-012. LPS stimulation increased superoxide generation in both sham- and CS-exposed mice, with a more pronounced response observed in the CS + LPS group (Figure 3E).

**FIGURE 4:**
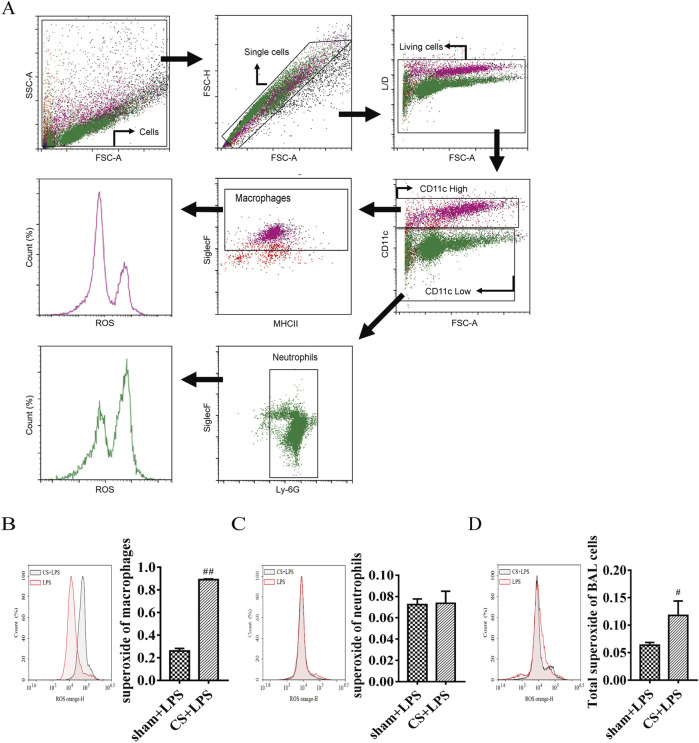
Cigarette smoke exposure exacerbates oxidative stress induced by lipopolysaccharide challenge in the lungs. **(A)** Gating strategy for the flow cytometric detection of macrophages and neutrophils in BAL fluid. **(B)** Flow cytometric measurement of macrophage superoxide production. **(C)** Flow cytometric measurement of neutrophil superoxide production. **(D)** Flow cytometric measurement of total superoxide production. Data are presented as mean ± SEM. Only the sham + LPS and CS + LPS groups were included in the flow cytometric ROS analysis (n = 12 and 14, respectively). #P < 0.05, ##P < 0.01, compared to sham + LPS mice.

To determine the cellular origin of excess superoxide production, flow cytometry was used to BAL cells (Figure 4A; Supplementary Table S1). As shown in Figure 4B, macrophage-derived superoxide production was significantly increased in the CS + LPS group, exhibiting a 3.4-fold elevation compared with sham + LPS controls. In contrast, neutrophil-derived superoxide levels were not significantly affected by CS exposure (Figure 4C). Consistently, total superoxide production was increased 1.85-fold in CS + LPS mice compared with sham + LPS mice (Figure 4D). These findings indicate that macrophages are the predominant source of ROS production in this model.

### Cigarette smoke exposure exacerbates IκBα phosphorylation induced by lipopolysaccharide challenge in lung tissue but does not promote apoptosis of pulmonary cells

**FIGURE 5:**
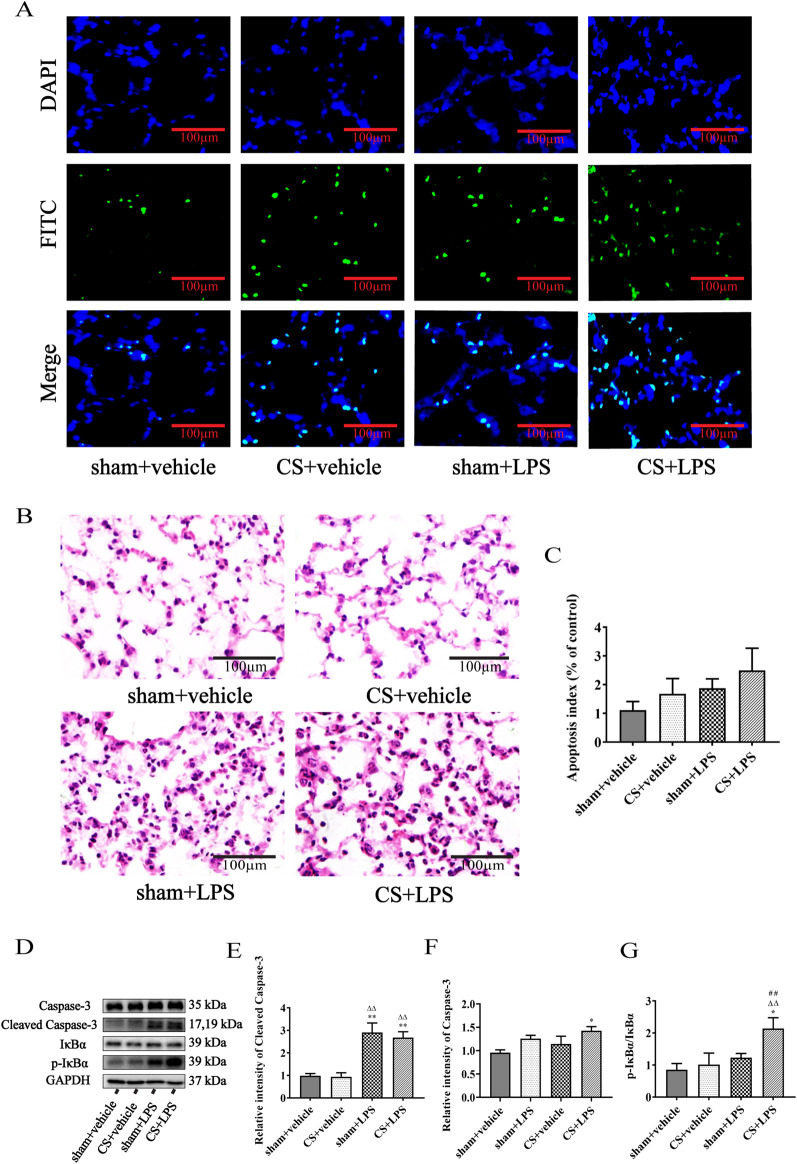
Cigarette smoke exposure exacerbates IκBα phosphorylation induced by lipopolysaccharide challenge in lung tissue but does not promote apoptosis of pulmonary cells. **(A)** Mouse pulmonary cell apoptosis was analyzed by TUNEL staining (original magnification, ×200). **(B)** Lung sections were subjected to H&E staining (original magnification, ×200). Five randomly selected, non-overlapping fields were examined per animal. Representative images from four experimental groups are shown. **(C)** Quantitative data are presented as the ratio of TUNEL-positive cells per examined area, n = 7. **(D)** Western blot analysis of protein expression of caspase-3, cleaved caspase-3, IκBα, and p-IκBα in lung tissue. GAPDH was used as the loading control. **(E–G)** Quantitative analyses of caspase-3, cleaved caspase-3, and the ratio of p-IκBα to IκBα, n = 6. Data are presented as mean ± SEM. *P < 0.05, **P < 0.01, compared to sham + vehicle mice; △P < 0.05, △△P < 0.01, compared to CS + vehicle mice; #P < 0.05, ##P < 0.01, compared to sham + LPS mice.

Pulmonary apoptosis was assessed using TUNEL staining. As shown in Figure 5A, LPS instillation increased the number of TUNEL-positive cells in lung sections from both sham- and CS-exposed mice. Histopathological analysis of H&E-stained lung sections revealed that LPS instillation induced inflammatory cell infiltration and thickening of the alveolar septa in both sham- and CS-exposed mice. No significant differences in the extent of these changes were observed between the sham + LPS and CS + LPS groups, as qualitatively evaluated from the representative images (Figure 5B). Quantitative analysis of TUNEL staining further confirmed that CS exposure did not significantly increase the apoptosis index in LPS-treated mice (Figure 5C). Western blot analysis of apoptosis- and inflammation-related proteins is shown in Figure 5D. Consistent with the TUNEL findings, LPS significantly elevated caspase-3 protein levels in both sham- and CS-exposed mice (Figure 5E). Similarly, cleaved caspase-3 expression increased following LPS instillation, but CS exposure did not further augment these apoptosis-related markers (Figure 5F). In contrast, inflammatory signaling was enhanced, as CS exposure combined with LPS instillation significantly increased IκBα phosphorylation compared to LPS treatment alone (Figure 5G), indicating amplified activation of NF-κB–related pathways.

### Cigarette smoke exposure enhances the expression of early remodeling-associated markers induced by lipopolysaccharide challenge in lung tissue

**FIGURE 6:**
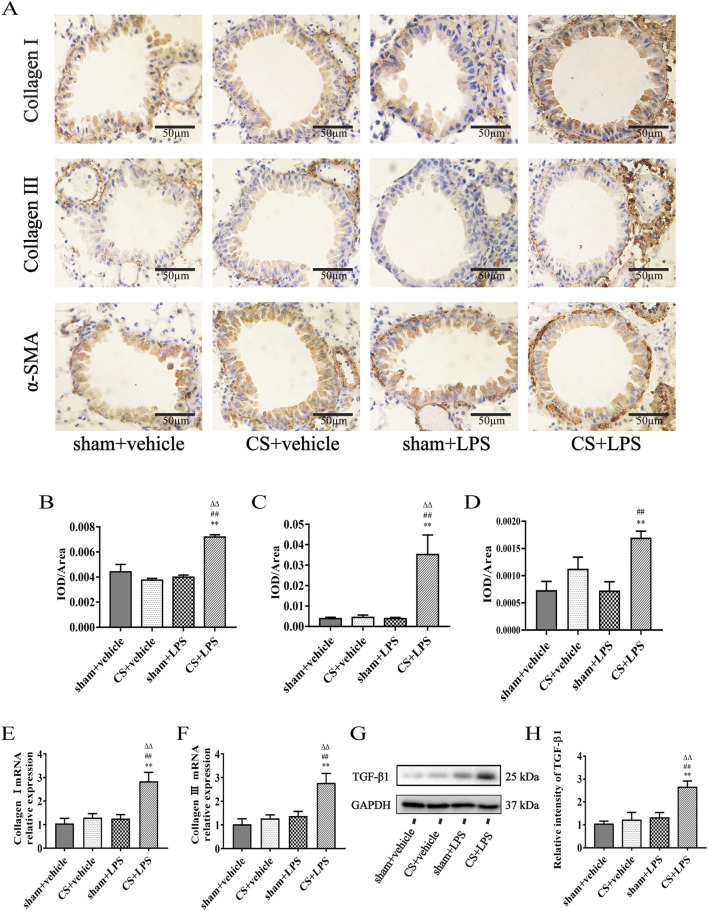
Cigarette smoke exposure enhances the expression of early remodeling-associated markers induced by lipopolysaccharide challenge in lung tissue. **(A)** Lung sections were subjected to immunohistochemical staining (original magnification, ×400). **(B–D)** Quantitative analysis of immunohistochemistry results for collagen I, collagen III, and α-SMA, based on the ratio of IOD to pulmonary parenchymal area, n = 4. **(E,F)** Quantitative real-time PCR (qPCR) analysis of collagen I and collagen III mRNA expression in lung tissue, n = 6–7. **(G)** Western blot analysis of TGF-β1 protein expression in lung tissue. GAPDH was used as the loading control. **(H)** Quantitative analysis of TGF-β1, n = 6. Data are presented as mean ± SEM. *P < 0.05, **P < 0.01, compared to sham + vehicle mice; △P < 0.05, △△P < 0.01, compared to CS + vehicle mice; #P < 0.05, ##P < 0.01, compared to sham + LPS mice.

To assess early remodeling-associated changes in the lung following exposure to cigarette smoke (CS) and lipopolysaccharide (LPS), we used immunohistochemistry to examine extracellular matrix markers (collagen I and collagen III) and the mesenchymal marker α-SMA. As shown in Figures 6A–D, exposure to CS or LPS alone did not induce detectable expression of collagen I, collagen III, or α-SMA. In contrast, combined CS exposure and LPS instillation resulted in a marked increase in these remodeling-related markers. Consistently, qPCR analysis revealed that CS exposure, combined with LPS instillation, significantly increased mRNA expression of collagen I and collagen III in lung tissue (Figures 6E,F). Moreover, Western blot analysis showed increased TGF-β1 protein expression in CS + LPS mice (Figures 6G,H), supporting activation of early remodeling-related signaling pathways.

## Discussion

The impact of long-term smoking on infection has been extensively studied, particularly in research on COPD. However, it remains unclear whether the infection response is affected in populations exposed to short-term cigarette smoke, such as intermittent smokers or passive smokers. This study examined the effects of 4 days of short-term cigarette smoke exposure on pulmonary immunity, lung tissue inflammation, injury, and repair following LPS instillation. The findings revealed that short-term cigarette smoke exposure did not induce significant oxidative stress or inflammatory responses, nor did it increase LPS-induced inflammatory cell recruitment. However, short-term cigarette smoke exposure exacerbated LPS-induced superoxide production in macrophages, intensified activation of pulmonary tissue inflammatory signaling pathways and levels of key oxidative stress enzymes, and amplified early airway remodeling signals.

Short-term cigarette smoke exposure has been reported to recruit immune cells into the airways and increase airway inflammation (Gualano et al., 2008; Yu et al., 2019), whereas long-term exposure can induce destruction of alveolar walls, leading to emphysema (Liu et al., 2023). Cigarette smoke contains a complex mixture of chemicals, including various reactive oxygen and nitrogen species, which can damage tissue and cellular components. In our study, short-term cigarette smoke exposure mildly increased BALF immune cell levels 1 day after cessation. It also slightly increased the levels of pro-inflammatory cytokines IL-6, IL-1β, and TNF-α, and mildly decreased the GSH/GSSG ratio. Additionally, there was a slight increase in the proportion of TUNEL-positive cells in lung tissue following short-term cigarette smoke exposure. Although short-term CS exposure did not produce significant changes relative to the control group, our data suggest that brief CS exposure acts as a “priming event,” consistent with previous studies (Gotts et al., 2017; Sakhatskyy et al., 2017; Cristaldi et al., 2023). The harmful substances in tobacco, along with their effects on lung immunity, inflammation, oxidative stress, and tissue damage, provide experimental evidence that short-term smoking alters the lung environment. These alterations underlie the exacerbation of LPS-induced NF-κB and TGF-β1/NOX2 pathways.

A key finding of this study is that smoking does not increase LPS-induced neutrophil and macrophage levels; however, it does elevate ROS in macrophages and exacerbate the expression of pro-inflammatory cytokines in lung tissue. This suggests that CS enhances the transcriptional inflammatory response without affecting immune cell recruitment (Anderson, 2008).

Interestingly, although there was no significant increase in neutrophil and macrophage levels in the CS + LPS group compared to the sham + LPS group, the neutrophil chemoattractants CXCL1 (3.9%) and CXCL2 (31.6%), as well as the macrophage chemoattractant MIP-1α (21.2%), were all elevated rather than suppressed in lung tissue. This observation suggests that, under the combined influence of cigarette smoke and LPS, abnormal neutrophil and macrophage death may occur. Our previous research also found that cigarette smoke extract can induce apoptosis and necrosis in neutrophils (Yu et al., 2019; Ruan et al., 2025). Phagocytosis of dying neutrophils by macrophages produces large amounts of superoxide, which rapidly lyses the engulfed cells (Park, 2003). This process may partly explain the significant increase in macrophage ROS levels.

High levels of ROS produced by macrophages exacerbate tissue irritation and damage, leading to the transcription of pro-inflammatory cytokines and activation of oxidative signaling pathways. Additionally, elevated ROS levels prevent macrophages from polarizing toward the M2 phenotype, keeping them in a compensatory M1 phenotype characterized by increased superoxide production. Consequently, lung tissue damage—including injury to epithelial and endothelial cells—is aggravated. As the balance between immune response and tissue repair is disrupted, lung tissue repair becomes increasingly reliant on non-M2 macrophage-mediated pathways. Accordingly, a significant increase in TGF-β1 induced by CS combined with LPS was observed in lung tissue, accompanied by localized collagen production within a short time frame.

Since short-term exposure to cigarette smoke significantly increased macrophage superoxide levels, we sought to investigate whether this change would trigger adverse pulmonary outcomes, including lung tissue damage and airway remodeling. However, histological analysis and apoptosis-related assays did not indicate that smoking exacerbated LPS-induced apoptosis or destruction of lung tissue cells. This finding is consistent with a previous report (Sakhatskyy et al., 2017). It suggests that although cigarette smoke contains substantial peroxide levels and induces excessive oxidative stress in macrophages, short-term exposure—even when combined with LPS—does not cause significant tissue damage, despite LPS activating the caspase-3 signaling pathway.

Interestingly, although lung tissue damage was not apparent, the smoke itself and the resulting excessive macrophage oxidative stress intensified the activation of lung tissue oxidative stress-related pathways, including TGF-β/NOX2 and the antioxidant enzyme HO-1. TGF-β is considered a key initiator and master regulator of tissue repair and wound healing (Patel et al., 2022; Deng et al., 2024). Accordingly, in this study, we observed increased collagen markers around the trachea and blood vessels in the CS + LPS group. However, these localized changes do not indicate that airway remodeling has fully developed, as collagen deposition is a dynamic process that requires repeated, long-term stimulation to accumulate gradually (Liu et al., 2021). The findings of this study suggest only that CS + LPS initiates airway remodeling-related activities and early release of pro-fibrotic signals. Nevertheless, this early fibrotic signaling may place individuals with intermittent smoke exposure at risk of chronic remodeling, especially if they experience recurrent infections or other stressors (Churg et al., 2006; Ganesan et al., 2014; Lee et al., 2018; Yang et al., 2021).

Overall, brief exposure to CS appears insufficient to cause overt oxidative damage, apoptosis, or structural injury on its own. Instead, it primes the pulmonary immune and redox environment, rendering the lung more sensitive to exaggerated inflammation, oxidative stress, and early fibrotic signaling upon subsequent microbial challenge. However, because dynamic changes in airway remodeling cannot be accurately captured at a single time point, it is impossible to rule out that the increased collagen deposition is transient. Therefore, whether temporary increases in collagen deposition truly affect the subsequent progression of airway remodeling remains unknown. The short-term CS exposure model was designed to mimic brief or intermittent exposure; however, it does not fully capture the complexity of real-world passive smoking conditions. Thus, caution is warranted when extrapolating these findings to human populations. Longer-term observation and mechanistic validation are required in future studies. Nevertheless, the results of this study suggest that brief exposure to cigarette smoke may cause excessive oxidative stress and immune damage during infection, highlighting the hazards of short-term cigarette smoke exposure and providing new insights into the initiating mechanisms of airway remodeling induced by long-term cigarette smoke exposure.

## Conclusion

Short-term exposure to cigarette smoke exacerbates LPS-induced reactive oxygen species production in macrophages, further aggravating inflammation, oxidative stress, and the expression of early airway remodeling markers through the NF-κB and TGF-β1/NOX2 pathways in lung tissue. These findings highlight the vulnerability of individuals exposed to cigarette smoke for a brief period to acute lung injury from bacterial challenges and reveal the potential harm of short-term cigarette smoke exposure during infection.

## Data Availability

The original contributions presented in the study are included in the article/Supplementary Material, further inquiries can be directed to the corresponding authors.
