# Correction: Short-term cigarette smoke exposure aggravates oxidative stress and airway inflammation induced by lipopolysaccharides

**DOI:** 10.3389/fphys.2026.1836865

**Published:** 2026-04-21

**Authors:** Ziyao Liang, Zhihang Liu, Wenchao Pan, Long Fan, Jingyu Quan, Lin Lin, Lei Wu, Xuhua Yu

**Affiliations:** State Key Laboratory of Traditional Chinese Medicine Syndrome, The Second Affiliated Hospital of Guangzhou University of Chinese Medicine/Guangzhou University of Chinese Medicine, Guangzhou, Guangdong, China

**Keywords:** cigarette smoke, inflammation, LPS, oxidative stress, airway remodeling

There was a mistake in **Figure 3A** as published. The molecular weight of “NOX2” was incorrectly displayed as “22 kDa”. The corrected **Figure 3A** appears below. The correct molecular weight of NOX2 is 58 kDa.

**Figure 3:**
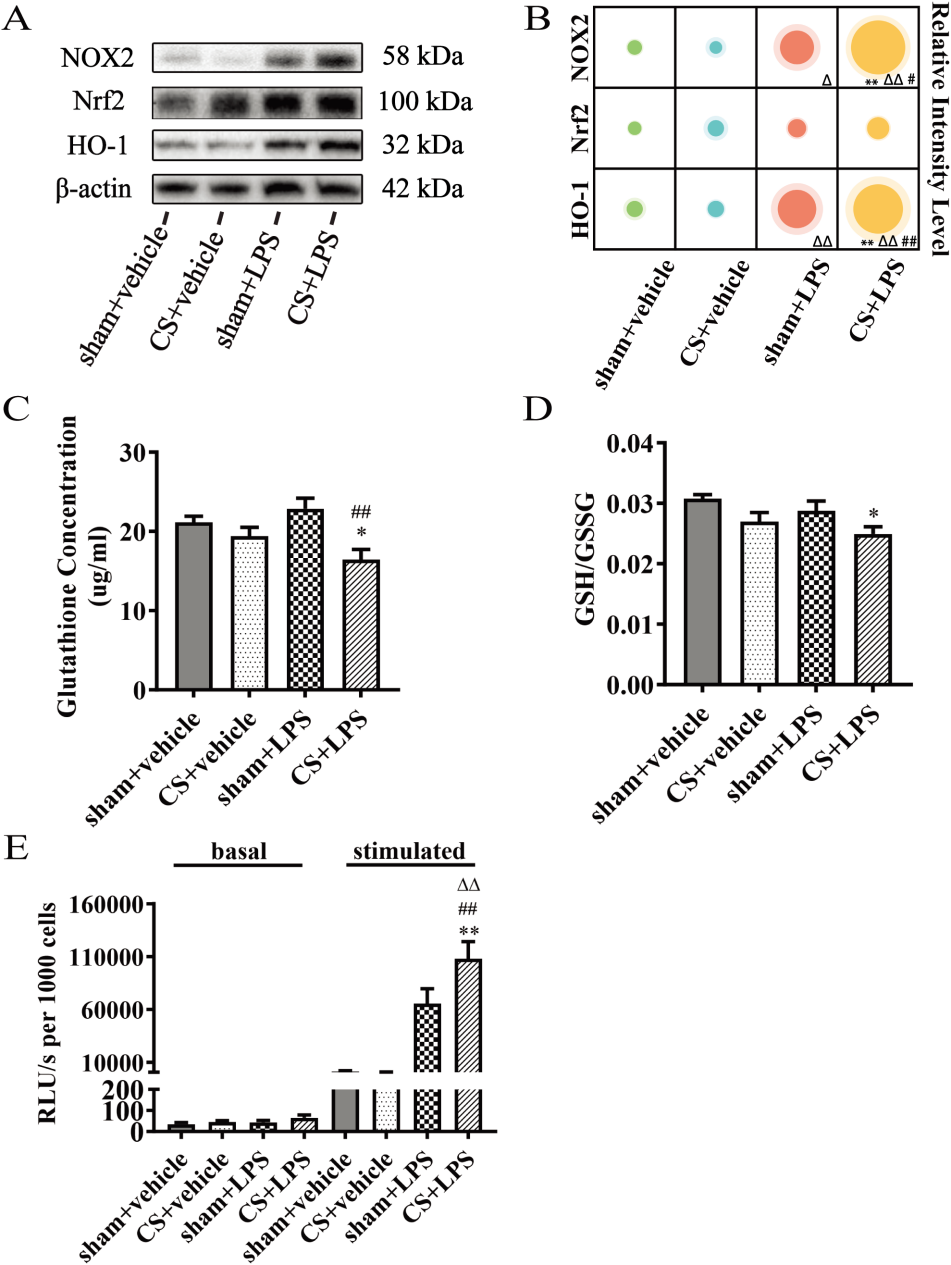
Cigarette smoke exposure amplifies the expression of inflammatory genes induced by lipopolysaccharide challenge in lung tissue. **(A)** Western blot analysis of protein levels of NOX2, Nrf2, and HO-1 in lung tissue. β-actin was used as the loading control. **(B)** Quantitative analysis of NOX2, Nrf2 and HO-1, n = 6. **(C)** Gutathione concentration, n = 7. **(D)** Ratio of GSH to GSSG, n = 7. **(E)** Superoxide production in BAL cells stimulated with PDBu was measured using the Multiscan Spectrum, n = 5–7. Data are presented as mean ± SEM. *P < 0.05, **P < 0.01, compared to sham + vehicle mice; △P < 0.05, △△P < 0.01, compared to CS + vehicle mice; #P < 0.05, ##P < 0.01, compared to sham + LPS mice.

There was a mistake in **Figure 5D** as published. The molecular weight of “p-IκBα” was incorrectly displayed as “35 kDa”. The corrected **Figure 5D** appears below. The correct molecular weight of “p-IκBα” is “39 kDa”.

**Figure 5:**
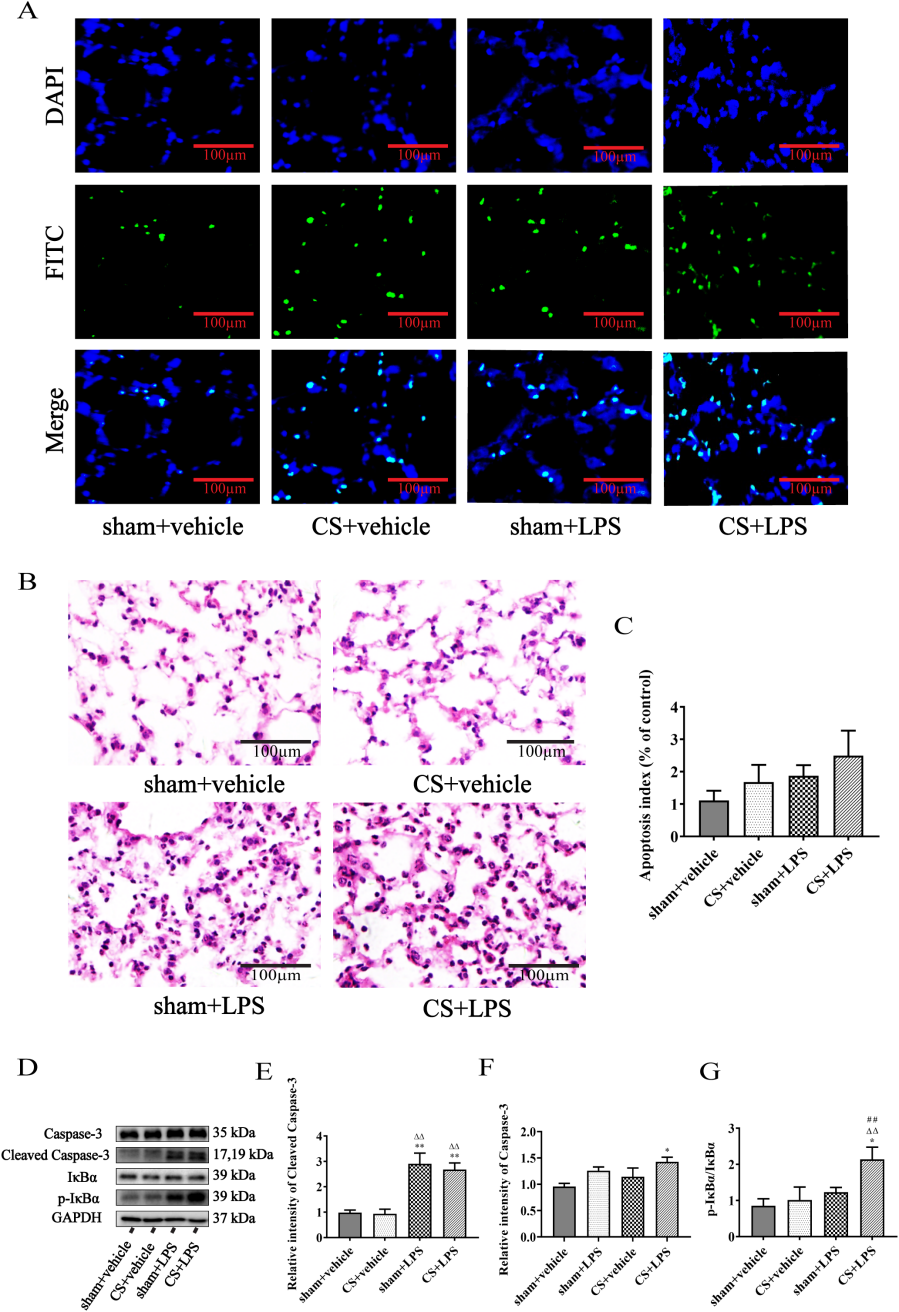
Cigarette smoke exposure exacerbates IκBα phosphorylation induced by lipopolysaccharide challenge in lung tissue but does not promote apoptosis of pulmonary cells. **(A)** Mouse pulmonary cell apoptosis was analyzed by TUNEL staining (original magnification, ×200). **(B)** Lung sections were subjected to H&E staining (original magnification, ×200). Five randomly selected, non-overlapping fields were examined per animal. Representative images from four experimental groups are shown. **(C)** Quantitative data are presented as the ratio of TUNEL-positive cells per examined area, n = 7. **(D)** Western blot analysis of protein expression of caspase-3, cleaved caspase-3, IκBα, and p-IκBα in lung tissue. GAPDH was used as the loading control. **(E–G)** Quantitative analyses of caspase-3, cleaved caspase-3, and the ratio of p-IκBα to IκBα, n = 6. Data are presented as mean ± SEM. *P < 0.05, **P < 0.01, compared to sham + vehicle mice; △P < 0.05, △△P < 0.01, compared to CS + vehicle mice; #P < 0.05, ##P < 0.01, compared to sham + LPS mice.

The original version of this article has been updated.

