# Supplementary material for: Short-term cigarette smoke exposure aggravates oxidative stress and airway inflammation induced by lipopolysaccharides
Source: Front Physiol. 2026 Mar 19;17:1788828. doi: 10.3389/fphys.2026.1788828 (PMC13043341; doi:10.3389/fphys.2026.1788828)
Supplement: Supplementary file 1 [file DataSheet1.pdf]

Supplementary Material

Supplementary Tables

Supplementary Table 1. Selection of Immune Cell Surface Antigens

Antigen-fluorophore to be added to cells	Antibody stock concentration (mg/mL)	Antibody dilution	Total volume (μL)
Fixable viability dye	0.2	1/1000	100
CD11c	0.125	1/800	100
SiglecF	1	1/200	100
MHCII	1	1/200	100
Ly6G	1	1/200	100

Supplementary Table 2. Primer sequence

Gene	Forward primer	Reverse primer
Chemokines		
Cxcl1	TGCACCCAAACCGAAGTCAT	CTCCGTTACTTGGGGACACC
Cxcl2	TCATAGCCACTCTCAAGGGC	TCAGGTACGATCCAGGCTTC
Cxcl5	CGGTTCCATCTCGCCATTCA	GCGGCTATGACTGAGGAAGG
Mip-1α	TAGCCACATCGAGGGACTCT	GATGGGGGTTGAGGAACGTG
Cytokines		
Il-6	ACAAAGCCAGAGTCCTTCAGAG	GAGCATTGGAAATTGGGGTAGG

Il-1β	GTCCTGTGTAATGAAAGACGGC	TGCTTGTGAGGTGCTGATGT
Tnf-α	ACGGCATGGATCTCAAGAC	GGAGGTTGACTTTCTCCTGGTA
Gm-csf	GCTCACTGGCCCCATGTATAG	GCTGAGAGGCTGTAGACCAC
Protease		
Mmp9	GCCCTGGAACTCACACGACA	TTGGAAACTCACACGCCAGAAG
collagen proteins		
Collagen Ⅰ	GAGAGGTGAACAAGGTCCCG	AAACCTCTCTCGCCTCTTGC
Collagen Ⅲ	CAAGGCTGCAAGATGGATGC	CAGTGCTTACGTGGGACAGT
